# Cost-effectiveness of ceftolozane/tazobactam compared with piperacillin/tazobactam as empiric therapy based on the in-vitro surveillance of bacterial isolates in the United States for the treatment of complicated urinary tract infections

**DOI:** 10.1186/s12879-017-2408-7

**Published:** 2017-04-28

**Authors:** Teresa L Kauf, Vimalanand S. Prabhu, Goran Medic, Rebekah H. Borse, Benjamin Miller, Jennifer Gaultney, Shuvayu S. Sen, Anirban Basu

**Affiliations:** 1 Shire International GmbH, Zug, Switzerland; 2 Merck & Co., Inc., Kenilworth, NJ USA; 3 MAPI Group, Houten, The Netherlands; 4 Shire, Lexington, MA USA; 5 University of Washington, Seattle, WA USA; 6 Merck & Co., Inc., 2000 Galloping Hill Rd., Kenilworth, NJ 07033 USA

**Keywords:** Cost-benefit analysis, Ceftolozane, Piperacillin, Tazobactam, Urinary tract infections, United States, Drug resistance

## Abstract

**Background:**

A challenge in the empiric treatment of complicated urinary tract infection (cUTI) is identifying the initial appropriate antibiotic therapy (IAAT), which is associated with reduced length of stay and mortality compared with initial inappropriate antibiotic therapy (IIAT). We evaluated the cost-effectiveness of ceftolozane/tazobactam compared with piperacillin/tazobactam (one of the standard of care antibiotics), for the treatment of hospitalized patients with cUTI.

**Methods:**

A decision-analytic Monte Carlo simulation model was developed to compare the costs and effectiveness of empiric treatment with either ceftolozane/tazobactam or piperacillin/tazobactam in hospitalized adult patients with cUTI infected with Gram-negative pathogens in the US. The model applies the baseline prevalence of resistance as reported by national in-vitro surveillance data.

**Results:**

In a cohort of 1000 patients, treatment with ceftolozane/tazobactam resulted in higher total costs compared with piperacillin/tazobactam ($36,413 /patient vs. $36,028/patient, respectively), greater quality-adjusted life years (QALYs) (9.19/patient vs. 9.13/patient, respectively) and an incremental cost-effectiveness ratio (ICER) of $6128/QALY. Ceftolozane/tazobactam remained cost-effective at a willingness to pay of $100,000 per QALY compared to piperacillin/tazobactam over a range of input parameter values during one-way and probabilistic sensitivity analysis.

**Conclusions:**

Model results show that ceftolozane/tazobactam is likely to be cost-effective compared with piperacillin/tazobactam for the empiric treatment of hospitalized cUTI patients in the United States.

**Electronic supplementary material:**

The online version of this article (doi:10.1186/s12879-017-2408-7) contains supplementary material, which is available to authorized users.

## Background

Gram-negative pathogens are a major cause of hospital-treated infections, accounting for 38% of all healthcare associated infections in the US [1–3]. Complicated urinary tract infections (cUTI), which are defined as UTIs associated with factors that compromise the urinary tract or host defense, are commonly caused by Gram-negative pathogens [4]. In the US, the prevalence of cUTI has been estimated at 24.2 per 1000 hospital discharges. Accounting for 70–80% of cUTIs [5], catheter-associated cUTIs make up a large group of cUTIs predominantly caused by Gram-negative pathogens [3, 6–8], including resistant pathogens, and reflect 28% of device-associated and procedure–associated infections [2, 9].

cUTI is often treated empirically as organism identification and susceptibility is not available at diagnosis. Patients with resistant pathogens are more likely to receive initially inappropriate antibiotic therapy (IIAT), defined as microbiological documentation of an infecting pathogen that was not effectively treated at the time of identification, instead of initially appropriate antibiotic therapy (IAAT) [10, 11]. Antibiotic resistance is associated with significant adverse impact on clinical outcomes, and increased consumption of health-care resources, leading to higher costs [12]. In a retrospective, matched-cohort analysis of patients admitted to the hospital with UTI in the US, patients with infections caused by extended-spectrum β-lactamase (ESBL) producing bacteria experienced IIAT 61.8% of the time as compared to 5.5% for patients with ESBL negative infections. Further, patients experienced two additional days in the hospital and an all-cause mortality rate of 9.1% with ESBL positive infections compared to 1.8% in ESBL negative infections. The increased length of stay and increased mortality in patients receiving IIAT vs. IAAT is also seen in other bacterial infections where antibiotics are administered for initial empiric therapy [13].

The goal of empiric therapy, therefore, is to increase the chances of IAAT. Thus, the antibacterial spectrum of the empiric antibiotic agent should cover the most relevant pathogens. Clinicians making decisions about empiric therapy for patients with cUTI not only consider the pathogens most likely colonizing the site of infection and knowledge of any prior bacteria known to colonize a given patient, but also local resistance patterns or antibiograms [14]. Local in-vitro antibiotic susceptibility data available through institutional antibiograms are more likely to reflect the current local resistance patterns compared with efficacy data from clinical trials alone as clinical trials are conducted internationally in geographically diverse populations. The application of local surveillance data for up-to-date clinical practice guidelines has been shown to be essential in patient care of cUTI given the evolving bacterial susceptibility [15].

Given the clinical and economic burden associated with IIAT, it is necessary to consider not only the clinical benefits but also the economic benefits that an empiric therapy could provide as a result of better coverage and improved susceptibility. There is a growing need to evaluate new and effective therapies that can offer a higher probability of appropriate empiric coverage compared to current antibacterial drugs.

In this study, we assess the cost-effectiveness of ceftolozane/tazobactam compared with piperacillin/tazobactam as empiric therapy in the treatment of hospitalized US patients aged 18 years or older with cUTI. Piperacillin/tazobactam is commonly used for empiric therapy of cUTI when resistance is suspected, as recommended in treatment guidelines [16, 17]. Ceftolazone/tazobactam is a novel cephalosporin/β-lactamase inhibitor combination available for treatment of adult cUTI patients. Ceftolazone/tazobactam has demonstrated broad activity against Gram-negative pathogens, including ESBL-producing Enterobacteriaceae and multi-drug resistant *Pseudomonas aeruginosa* [18].

## Methods

### Model structure

**Fig. 1:**
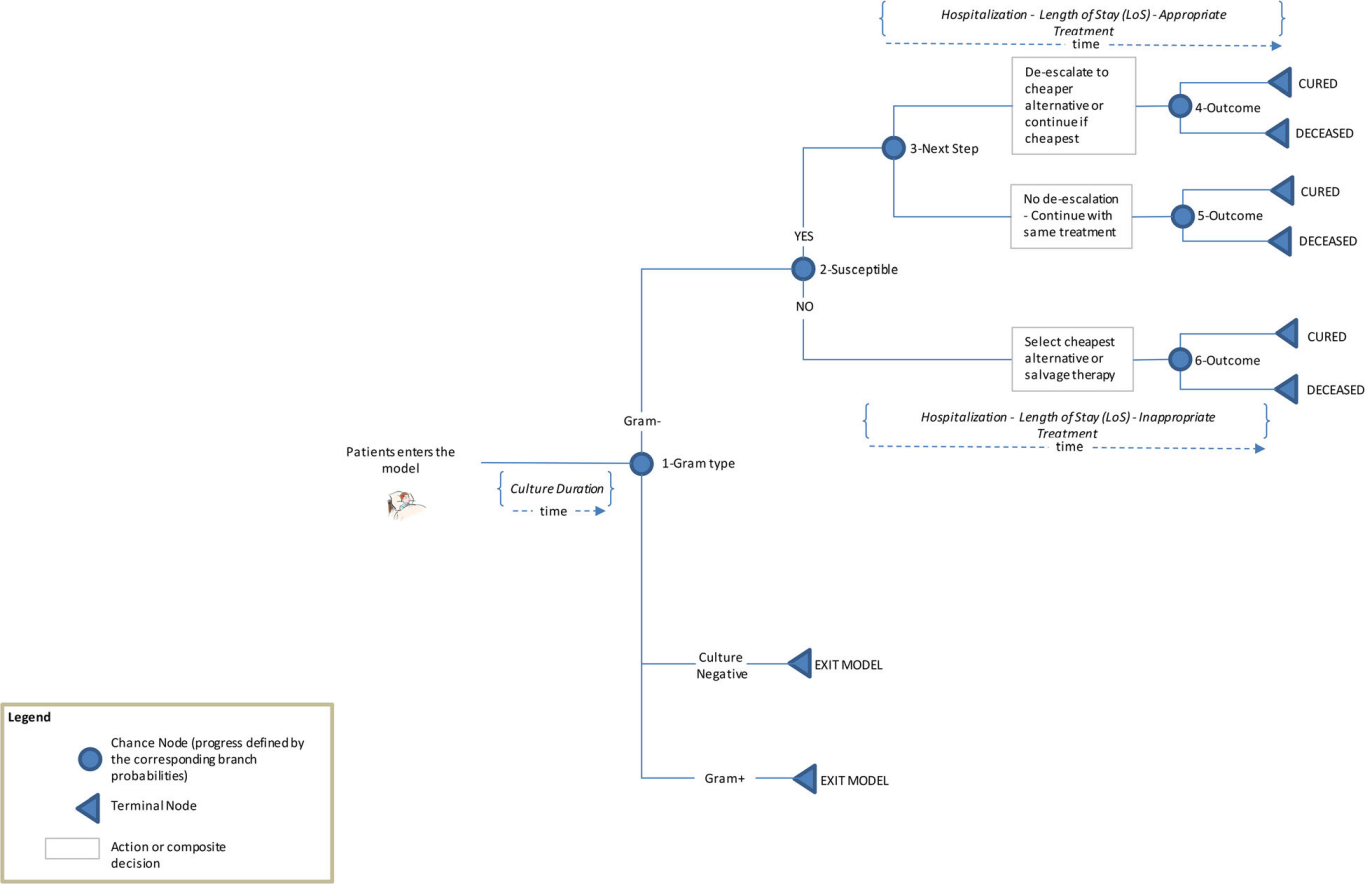
Model structure

A patient-level decision analytic Monte Carlo micro-simulation model was developed to estimate the quality-adjusted life expectancy and costs of persons diagnosed with cUTI in order to conduct a cost-utility analysis of ceftolozane/tazobactam compared with piperacillin/tazobactam in the target patient population. The model tracks index patients through different phases of cUTI from diagnosis until death. A graphical representation of the model structure with all treatment pathways is provided in Fig. 1. The model incorporates treatment switching algorithms that depend upon patient level data regarding the underlying pathogen and its susceptibility to various antibiotics. As a result, an individual patient simulation that captures patient level information and history is a more appropriate model as opposed to a Markov model with transition probabilities that do not depend on history.

Hospitalized patients enter the model at the time of cUTI diagnosis, which is assumed to be concurrent with collecting the urine culture and initiation of empiric antimicrobial therapy. Each patient in the model receives empiric antibiotic treatment with either ceftolozane/tazobactam or piperacillin/tazobactam. Patients continue empiric treatment until culture results are available (assumed to occur after 3 days). Culture results include organism identification and susceptibility for ceftolozane/tazobactam, piperacillin/tazobactam, and other drugs that patients could be given, consistent with standard treatments. Once culture results are known, patients are switched to the least expensive therapy to which the causative pathogen is susceptible. Patients may be maintained on the same drug (initial drug is appropriate and cheapest), de-escalated to a cheaper drug (initial drug is appropriate, but another drug that is cheaper and to which the pathogen is susceptible is available), or escalated (initial therapy is inappropriate, another drug to which the pathogen is susceptible is available, with patient escalated to the cheapest drug to which the pathogen is susceptible). If the pathogen is not susceptible to any of the drugs for which culture results were obtained, patients are switched to salvage therapy, assumed in this analysis as the combination of meropenem and colistin. Patients with Gram-positive infections exit the model after 3 days of initial therapy (Fig. 1). As the underlying pathogen is unknown at diagnosis, patients suspected of a Gram-negative infection but infected with a Gram-positive pathogen may be inadvertently prescribed drugs for the treatment of a Gram-negative infection. While empiric therapy with these drugs adds to the cost, it does not have any impact on the resolution of symptoms. As both ceftolozane/tazobactam and piperacillin/tazobactam are not indicated for Gram-positive pathogens, we assume that disease progression, after determination of culture results, and therefore the subsequent costs and outcomes, are similar across both arms. Drug acquisition costs until culture results become available are included; however, we did not include any other costs as they are likely to be same for both arms.

The patient-level simulation begins with the random selection of an isolate from the Program to Assess Ceftolozane/Tazobactam Susceptibility (PACTS) surveillance dataset [19], such that each isolate represents a single patient. Isolates were sampled so that the resulting pathogen distribution reflects that of cUTI, as determined by an analysis of Premier hospital discharge data. Details regarding PACTS and Premier data are provided in Additional File 1.

Each patient enters the decision tree, and relevant costs are accumulated as they progress through one of the treatment pathways. This process is repeated for a large number of patients, and corresponding results are used to produce point estimates and confidence intervals for total costs per comparator. The analysis was performed on a cohort of 1000 patients, with the patients for each comparator arm being identical. The appropriateness of initial antibiotic therapy influences each patient’s length of hospital stay. Once the patient finishes the entire duration of antibiotic therapy, mortality rate is dependent on whether the patient received IAAT or IIAT.

For patients who survive, we assume that they experience an average length of life based on their life expectancy and incur health care expenditure comparable to the average of a person their age [20].

### Interventions

The empiric treatment comparators considered in the model are ceftolozane/tazobactam and piperacillin/tazobactam. The following drugs were considered for switching (escalation/de-escalation) upon pathogen confirmation: aztreonam, cefepime, ceftazidime, ceftriaxone, ciprofloxacin, doripenem, imipenem, levofloxacin, meropenem and tigecycline.

### Time horizon, discounting and payer perspective

A lifetime horizon is applied to capture the utility and costs of healthy survivors. Costs and outcomes are discounted at 3% [21]. A US healthcare perspective is taken to evaluate costs.

### Clinical inputs

Susceptibility data from the PACTS dataset were used to evaluate the appropriateness of the treatments in the model (Additional file 1). Five-percent of patients were Gram positive based on the Phase-III trial results [22]. The other key clinical inputs are summarized in Table 1.

**Table 1:** Model inputs

Mortality rates	Mean	Lower bound	Upper bound	Distribution for PSA	Source
Mortality rate with appropriate empiric treatment	0.018	0.016	0.020	Beta	MacVane et al. [23]
Mortality rate with inappropriate empiric antibiotic	0.072	0.065	0.079	Beta	MacVane et al. [23]
Duration of therapy	Mean	Lower bound	Upper bound	Distribution for PSA	Source
Duration of empiric therapy	3 days	3 days	3 days	Gamma	MacVane et al. [23]
Total LOS for IAAT (inc. empiric therapy)	4 days	3 days	6 days	Gamma	MacVane et al. [23]
Additional LOS associated with IIAT	2 days	1 days	2 days	Gamma	MacVane et al. [23]
Quality of life adjustment	Mean	Lower bound	Upper bound	Distribution for PSA	Source
Health utility for survivors	0.85	0.70	1.00	Beta	Assumption
Hospital costs	Mean	Lower bound	Upper bound	Distribution for PSA	Source
Hospital cost per day (average USD 2015)	$1746.27	$1397.01	$2095.52	Gamma	AHQR [35]
Discounting	Mean	Lower bound	Upper bound	Distribution for PSA	Source
Benefits discount rate (per annum)	3%	3%	3%	Gamma	AMCP [21]
Drug name	Cost per day (USD 2015)				Source
Ceftolozane/tazobactam	$249.00				Analy$ource database [1]
Aztreonam	$84.24				
Cefepime	$23.04				
Ceftazidime	$19.80				
Ceftriaxone	$6.40				
Ciprofloxacin	$5.26				
Doripenem	$125.22				
Imipenem	$73.12				
Levofloxacin	$6.24				
Meropenem	$81.51				
Piperacillin/tazobactam	$43.08				
Tigecycline	$238.44				
Salvage^a^	$164.31				
Lifetime health care expenditure	Annual cost				Source
<25 years	$477				Basu [20]
25 to 34 years	$790				Basu [20]
35 to 44 years	$947				Basu [20]
45 to 54 years	$1422				Basu [20]
55 to 64 years	$2106				Basu [20]
65 to 74 years	$2758				Basu [20]
75 years and above	$3100				Basu [20]

*LOS* Length of stay, *IAAT* Initial appropriate antibiotic therapy, *IIAT* Initial inappropriate antibiotic therapy

^a^Salvage therapy consists of meropenem + colistin for cost purposes

Mortality rates and length of stay were based on MacVane et al. (2014) [23]. Duration of empiric therapy was assumed to be 3 days. US life-tables were used for the prediction of life expectancy (according to gender) [24].

### Economic inputs

Hospitalization costs per day were derived from the 2013 Healthcare Cost and Utilization Project (HCUP), based on primary diagnoses of urinary tract infection (ICD-9 code 590.10, 590.11, 590.2, 590.3, 590.80, 590.81, 590.9, 597, and 599) and catheter/device-associated infections (ICD-9 codes 996.64 and 996.65) [25]. The average cost per hospital day for cUTI patients, inflated to 2015 using the Gross Domestic Product (GDP) price index, was $1746.27.

Daily drug costs were based on wholesale average cost at labeled doses [26]. Salvage therapy costs were based on combination therapy with meropenem and colistin.

For healthy survivors, lifetime health care expenditure was calculated using average annual age-adjusted values [20] inflated to 2015 values using the Gross Domestic Product (GDP) price index (Table 1) [27].

A utility value of 0.85 (assumption) was applied to cured patients for the remainder of their lives (Table 1) [28].

### Analysis

The model compared ceftolozane/tazobactam with piperacillin/tazobactam from the perspective of the US hospital payer. The population was restricted to the US PACTS dataset for all available isolates from years 2011 to 2013. The susceptibility evaluation used Clinical and Laboratory Standards Institute (CLSI) breakpoints. A susceptibility breakpoint of 2 mg/L for all pathogens was assumed for ceftolozane/tazobactam, except for *Pseudomonas* spp. where a susceptibility breakpoint of 4 mg/L was used [29].

To compare the two treatment strategies, the following outcomes were estimated from the model: proportions of patients appropriately and inappropriately treated (sensitive/resistant to empiric therapy), cost per QALY saved, drug costs, hospitalization costs, proportion of cases by pathogen, total costs (undiscounted), and total QALYs (undiscounted and discounted). Differences in these outcomes of interest were estimated, along with the incremental cost-effectiveness ratio (ICER) calculated as total incremental cost per incremental QALY gained.

In order to evaluate uncertainty, one-way sensitivity analyses (OWSA) and probabilistic sensitivity analysis (PSA) were performed.

The model assessed the sensitivity of the model results to all the input data for which uncertainty has been defined one parameter at a time by means of OWSA. Beta distributions for utilities, gamma distributions for resource use and costs, reported statistical measures of uncertainty, where available, and otherwise within a range of ±10%. The ten parameters with the greatest impact were summarized in a tornado diagram.

For the PSA, new model input parameter values were repeatedly sampled from the defined distributions and the corresponding model output was calculated. The output values of 10,000 parameter samples were calculated to reflect the uncertainty in model output given the uncertainty of the input parameters.

For each treatment strategy, the probability of cost-effectiveness was expressed with cost-effectiveness acceptability curves, calculated as the number of iterations out of the total number of iterations for which the net monetary benefit (NMB) was greatest for a given treatment strategy out of all strategies. The NMB was calculated as the QALYs multiplied by a willingness to pay (WTP) ratio minus the costs, where a WTP of $100,000 was the amount decision makers were assumed to be willing to pay per additional QALY gained [30].

### Scenario analyses

Appropriate use of carbapenems is important as they are often used as the last line of defence against increasingly difficult-to treat Gram-negative pathogens. In the base case, the de-escalation algorithm ensures that patients continue treatment on the least expensive antibiotic to which an isolate is susceptible following the empiric therapy period. However, an alternative algorithm, the carbapenem-sparing option, was designed to evaluate the impact of the de-escalation algorithm on the cost-effectiveness results. In this scenario, a carbapenem therapy (doripenem, imipenem or meropenem) is selected only if there is no other treatment alternative to which the pathogen is susceptible. Even if a carbapenem was the cheapest agent available to which the pathogen was susceptible, it would not be used if another non-carbapenem (e.g., a cephalosporin) was available.

Two additional scenarios were designed to evaluate the impact of risk factors associated with infection due to resistant pathogens, as identified in the literature [31, 32]. Information regarding the risk factors for infection due to resistant pathogens in cUTI was available for patients in the PACTS dataset, including (a) nosocomial infection, (b) age ≥ 65 years, and (c) admission to the intensive care unit (ICU). Scenario analyses were performed firstly using only nosocomial isolates and secondly using only nosocomial isolates for high risk patients aged ≥65 years, requiring an ICU stay or experiencing a catheter-associated infection.

Lastly, an additional scenario was also performed where lifetime health care expenditure for healthy survivors was excluded.

## Results

### Base case results

The average age of the cohort was 75.1 years. Distribution of the major Gram-negative pathogens was as follows: 58% *Escherichia coli*, 18% *Klebsiella pneumoniae*, 10% *Psuedomonas aeruginosa*, and 8% *Proteus mirabilis*.

The key results from the model are summarized in Table 2. In the base case, ceftolozane/tazobactam resulted in higher total costs than piperacillin/tazobactam ($36,413/patient vs. $36,028/patient, respectively), a greater number of discounted QALYs (9.19/patient vs. 9.13/patient, respectively) and 249 hospitalization days saved.

**Table 2:** Summary of results

	Ceftolozane/tazobactam	Piperacillin/tazobactam	Incremental Ceftolozane/tazobactam - Piperacillin/tazobactam
Total costs per patient (USD 2015)	$36,413	$36,028	$385
Total QALYs (undiscounted) per patient	11.82	11.74	0.08
Total QALYs (discounted) per patient	9.19	9.13	0.06
Incremental Cost Effectiveness Ratio (Cost per discounted QALY gained)			$6128
Hospitalization days saved per patient			0.25

*QALY* Quality-adjusted life year

In patients receiving ceftolozane/tazobactam, 7.8% of pathogens were resistant compared with 20.2% of pathogens in those receiving piperacillin/tazobactam Table 3. There were 22.2 deaths (2.2%) in patients treated with ceftolozane/tazobactam compared with 28.9 (2.9%) in those treated with piperacillin/tazobactam. Amongst those who died, a larger proportion was resistant to initial therapy with piperacillin/tazobactam compared with ceftolozane/tazobactam.

**Table 3:** Appropriateness of empiric therapy

	Ceftolozane/tazobactam	Piperacillin/tazobactam
Resistant to initial therapy (%)	7.8	20.2
Susceptible to initial therapy (%)	92.2	79.8

When examining results for QALYs in more detail, ceftolozane/tazobactam generated a total of 0.06 discounted additional QALYs per patient compared with piperacillin/tazobactam. The average QALYs gained by patients treated with ceftolozane/tazobactam and piperacillin/tazobactam was 9.19 versus 9.13 (discounted), respectively.

Per patient lifetime health care expenditure and per patient drug costs were higher for ceftolozane/tazobactam compared with piperacillin/tazobactam ($28,651 vs. $28,444 and $766 vs. $155, respectively) Table 4. These were partly offset by hospital costs, with a lower average hospital cost per patient for patients treated with ceftolozane/tazobactam compared with those treated with piperacillin/tazobactam ($6996 vs. $7429, respectively). The resultant total cost per patient was higher for ceftolozane/tazobactam compared with piperacillin/tazobactam ($36,413 vs. $36,028, respectively).

**Table 4:** Cost results (USD 2015)

	Ceftolozane/tazobactam	Piperacillin/tazobactam	Incremental Ceftolozane/tazobactam - Piperacillin/tazobactam
Hospital costs per patient	$6996	$7429	-$433
Drug costs per patient	$766	$155	$612
Lifetime health care expenditure per patient	$28,651	$28,444	$207

For ceftolozane/tazobactam, 99.8% of patients who received IAAT were de-escalated after 3 days (following culture results), which was higher compared with piperacillin/tazobactam at 97.0%. In patients who received IIAT, an equal percentage of patients for each comparator (1.6%) required salvage therapy with meropenem + colistin.

### Sensitivity analyses

**Fig. 2:**
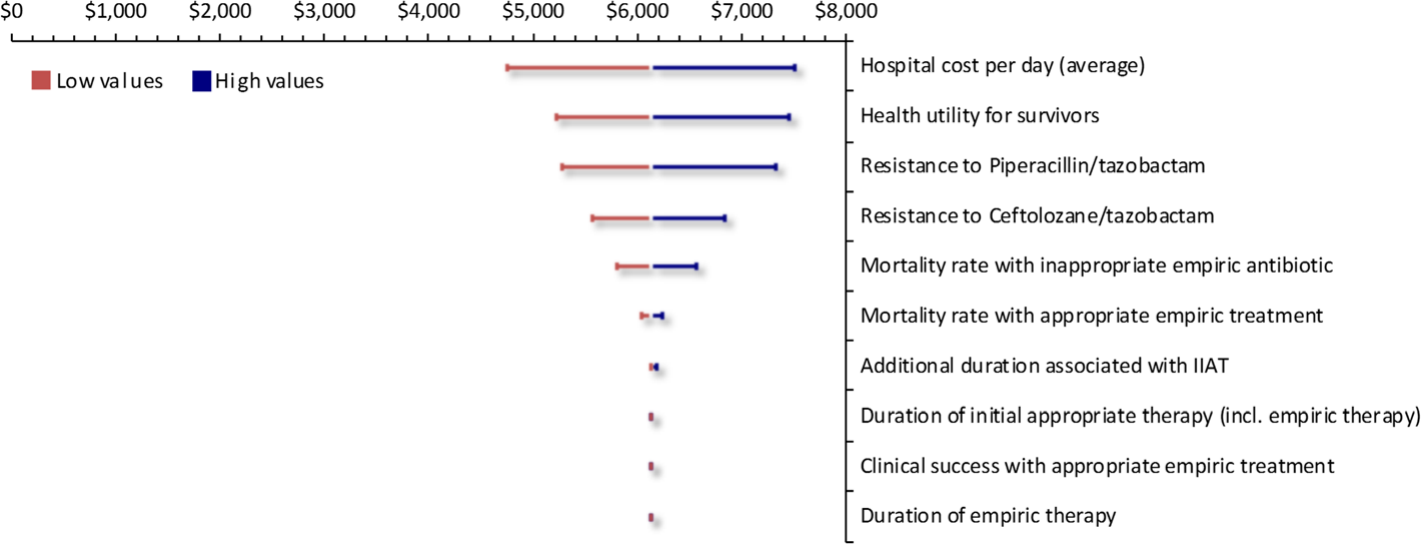
Ceftolozane/tazobactam vs. piperacillin/tazobactam: Tornado diagram illustrating influence of variables on ICER (cost per discounted QALY). ICER: Incremental cost-effectiveness ratio; LOS: Length of stay; QALY: Quality-adjusted life year

The results of the one-way sensitivity analysis are presented in a tornado graph (Fig. 2). Varying the average cost per hospital day resulted in the largest impact on the resultant ICER. Other input parameters influencing the model results included: the health utility value applied to survivors, susceptibilities, mortality rate associated with IIAT and IAAT, and the additional length of stay associated with IIAT.

**Fig. 3:**
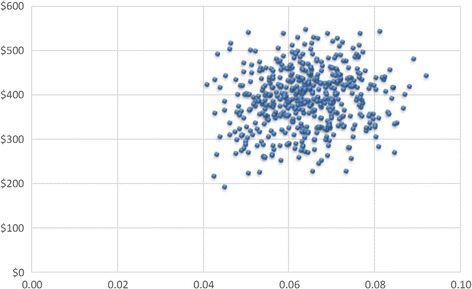
Cost-effectiveness plane. X-axis: Incremental QALYs, Y-axis: Incremental costs (USD), QALY: Quality-adjusted life year

The PSA shows that in all instances, ceftolozane/tazobactam is more effective and more costly than piperacillin/tazobactam (Fig. 3); however, ceftolozane/tazobactam has a 100% probability of being cost-effective at a willingness-to-pay threshold of $100,000/QALY gained.

### Scenario analyses

Table 5 provides the results of the scenario analyses. In all scenarios, ceftolozane/tazobactam resulted in higher total costs and a greater number of discounted QALYs than piperacillin/tazobactam. The carbapenem-sparing scenario and the scenario using only nosocomial isolates in high risk patients (aged ≥65 years, ICU stay, or catheter-associated infection) both resulted in ICERs that were very similar to the base case ($6020/QALY and $6037/QALY, respectively). Whilst the ICERs for the scenario using only nosocomial isolates (25% of the patients from the US PACTS dataset) and the scenario where lifetime health care expenditure was excluded were lower than in the base case ($3825/QALY and $2842/QALY, respectively).

**Table 5:** Scenario analysis results

Results for carbapenem-sparing scenario where non-carbapenem drugs are given precedence	Ceftolozane/tazobactam	Piperacillin/tazobactam	Incremental Ceftolozane/tazobactam - Piperacillin/tazobactam
Total costs per patient (USD 2015)	$36,416	$36,038	$378
Total QALYs (discounted) per patient	9.19	9.13	0.06
Incremental Cost Effectiveness Ratio (Cost per discounted QALY gained)			$6020
Results using only nosocomial isolates	Ceftolozane/tazobactam	Piperacillin/tazobactam	Incremental Ceftolozane/tazobactam - Piperacillin/tazobactam
Total costs per patient (USD 2015)	$42,737	$42,358	$378
Total QALYs (discounted) per patient	12.37	12.27	0.10
Incremental Cost Effectiveness Ratio (Cost per discounted QALY gained)	-	-	$3825
Results for high risk patients (aged 65 years, requiring an ICU stay or catheter-associate infection) using nosocomial isolates	Ceftolozane/tazobactam	Piperacillin/tazobactam	Incremental Ceftolozane/tazobactam - Piperacillin/tazobactam
Total costs per patient (USD 2015)	$37,947	$37,557	$390
Total QALYs (discounted) per patient	10.10	10.03	0.07
Incremental Cost Effectiveness Ratio (Cost per discounted QALY gained)	-	-	$6037
Results when lifetime health care expenditure for health survivors is excluded	Ceftolozane/tazobactam	Piperacillin/tazobactam	Incremental Ceftolozane/tazobactam - Piperacillin/tazobactam
Total costs per patient (USD 2015)	$7762	$7583	$179
Total QALYs (discounted) per patient	9.19	9.13	0.06
Incremental Cost Effectiveness Ratio (Cost per discounted QALY gained)	-	-	$2842

*QALY* Quality-adjusted life year

## Discussion

The objective of this analysis was to evaluate the use of ceftolozane/tazobactam compared with piperacillin/tazobactam in the empiric treatment of adult US patients with cUTI at risk of infection due to a resistant Gram-negative pathogen. Model results suggest that the use of ceftolozane/tazobactam as empiric treatment is cost-effective compared with piperacillin/tazobactam. Ceftolozane/tazobactam was associated with a higher proportion of patients with IAAT, resulting in reduced hospitalizations and increased QALYs.

The present study is the first economic evaluation of ceftolozane/tazobactam compared to the standard of care in the treatment of cUTI. The benefits of using ceftolozane/tazobactam as empiric therapy in this study are predominantly due to the proportion of the isolates that are susceptible to this therapy, as compared to the reference therapy. Therefore, the source and reliability of the susceptibility data are important. A strength of this study is the use of real-world surveillance data for the US from the PACTS surveillance database rather than clinical trial data. A similar approach to ours was used in the study by Sader et al. 2007 where they used the SENTRY Antimicrobial Surveillance Program, a large multinational data source on pathogen prevalence and antimicrobial susceptibility, to estimate the effectiveness of tigecycline in complicated skin and skin structure infections [33]. The findings of Sader et al. 2007 demonstrated the variation in prevalence of bacterial pathogens, highlighting the need to take into account local data on both frequency and susceptibility patterns [33]. Therefore, it is important to note that antimicrobial resistance varies by location, which could impact the cost-effectiveness of ceftolozane/tazobactam compared to piperacillin/tazobactam. For example, a recent study by Lin et al. 2015 compared the costs and effectiveness of ceftriaxone, ertapenem, and levofloxacin in treatment of community-acquired complicated urinary tract infections from a single center perspective [34]. Future studies evaluating the cost-effectiveness of ceftolozane/tazobactam applying local surveillance data are warranted.

A few limitations to the study deserve mention. Firstly, the PACTS dataset does not contain enough information to specifically target (a) cUTI patients and (b) all patients in the PACTS dataset at risk for resistant infection. Therefore, the true proportion of resistant pathogens in the target cohort and, consequently, the cost-consequence analysis of ceftolozane/tazobactam may have been underestimated. Secondly, the model does not account for further treatment changes after any initial de-escalation/escalation, with patients assumed to be fully cured or deceased at the end of hospitalization. Recurrence and/or re-admission were not incorporated in this model. Additionally, the model excludes bacterial resistance over time and costs of antibiotic preparation and administration, monitoring, and adverse events. These costs were assumed to be similar across treatments and/or minor. Similarly, dose adjustments were not considered.

The PACTS database includes monomicrobial infections; however, infections can be polymicrobial. Underlying polymicrobial susceptibility data are required to accurately model polymicrobial infections. We used the Premier database, which is a polymicrobial database, to define the distribution for our underlying pathogens. The sum of pathogens from the Premier study exceeded 100% as patients could have polymicrobial infections. As the model only considers monomicrobial infection, the Premier distributions had to be normalized to 100%.While our model is a first step in that direction and defines a way to model monomicrobial infections, more data are needed on polymicrobial infections to accurately model such infections.

The costs reported in this analysis may be overestimated since the isolates in PACTS may under-represent pathogen resistance in the target population of cUTI patients at risk of resistant infection. Costs are a function of several model parameters including duration of empiric therapy, susceptibility among comparators, and particularly the increase in length of stay (LOS) due to IIAT. Furthermore, the differences in costs are derived solely from differences in antimicrobial activity between ceftolozane/tazobactam and piperacillin/tazobactam.

The results of our analysis were largely unchanged in both the high risk scenarios and the carbapenem-sparing scenario, with ceftolozane/tazobactam remaining similarly cost-effective. Due to increasing antibacterial resistance and scarcity of new classes of antibacterial drugs to treat Gram-negative bacteria, it is necessary to preserve the efficacy of existing drugs to cure common and life-threatening infections [12].

The exclusion of lifetime health care expenditures in our base case analysis approximately halved the incremental costs, resulting in a lower ICER. For our analysis, ceftolozane/tazobactam remained cost-effective; however, inclusion of lifetime healthcare expenditure may have a potential impact on comparisons which are borderline cost-effective or cost-saving.

## Conclusion

Model-based analysis indicate that ceftolozane/tazobactam is cost-effective compared with piperacillin/tazobactam for the empiric treatment of cUTI in hospitalized patients.

## Additional file

Additional file 1:
*Details regarding PACTS and Premier data*. This additional file provides readers with additional details regarding the Program to Assess Ceftolozane/Tazobactam Susceptibility (PACTS) dataset which was used to provide susceptibility inputs for the model and also regarding the Premier research database which was used to help define the pathogen distribution in cUTI. (DOCX 29 kb)

## Abbreviations

CLSI: Clinical and Laboratory Standards Institute
cUTI: Complicated urinary tract infection
ESBL: Extended-spectrum β-lactamase
GDP: Gross Domestic Product
HCUP: Healthcare Cost and Utilization Project
IAAT: Initial appropriate antibiotic therapy
ICD-9: International Classification of Diseases, Ninth Edition
ICER: Incremental cost-effectivness ratio
IIAT: Initial inappropriate antibiotic therapy
LOS: Length of stay
NMB: Net monetary benefit
OWSA: One-way sensitivity analyses
PACTS: Program to Assess Ceftolozane/Tazobactam Susceptibility
PSA: Probabilistic sensitivity analysis
QALYs: Quality-adjusted life years
US: United States

## References

[1] Allegranzi B, Bagheri Nejad S, Combescure C, Graafmans W, Attar H, Donaldson L, Pittet D (2011). Burden of endemic health-care-associated infection in developing countries: systematic review and meta-analysis.

[2] Sievert DM, Ricks P, Edwards JR, Schneider A, Patel J, Srinivasan A, Kallen A, Limbago B, Fridkin S, National Healthcare Safety Network T (2013). Antimicrobial-resistant pathogens associated with healthcare-associated infections: summary of data reported to the National Healthcare Safety Network at the Centers for Disease Control and Prevention, 2009-2010. Infection control and hospital epidemiology: the official journal of the Society of Hospital Epidemiologists of America.

[3] European Centre for Disease Prevention and Control (ECDC) (2011). Antimicrobial resistance surveillance in Europe.

[4] Flores-Mireles AL, Walker JN, Caparon M, Hultgren SJ (2015). Urinary tract infections: epidemiology, mechanisms of infection and treatment options. Nat Rev Microbiol.

[5] Lo E, Nicolle LE, Coffin SE, Gould C, Maragakis LL, Meddings J, Pegues DA, Pettis AM, Saint S, Yokoe DS (2014). Strategies to prevent catheter-associated urinary tract infections in acute care hospitals: 2014 update. Infection control and hospital epidemiology: the official journal of the Society of Hospital Epidemiologists of America.

[6] Food and Drug Administration (FDA), Center for Drug Evaluation and Research (CDER) (2012). Guidance for industry. Complicated urinary tract infections: Developing drugs for treatment.

[7] Davis N, Flood H (2011). The pathogenesis of urinary tract infections.

[8] Nicolle LE, Louie TJ, Dubois J, Martel A, Harding GK, Sinave CP (1994). Treatment of complicated urinary tract infections with lomefloxacin compared with that with trimethoprim-sulfamethoxazole. Antimicrob Agents Chemother.

[9] Hidron AI, Edwards JR, Patel J, Horan TC, Sievert DM, Pollock DA, Fridkin SK, National Healthcare Safety Network T, Participating National Healthcare Safety Network F (2008). NHSN annual update: antimicrobial-resistant pathogens associated with healthcare-associated infections: annual summary of data reported to the National Healthcare Safety Network at the Centers for Disease Control and Prevention, 2006-2007. Infect Control Hosp Epidemiol.

[10] Kollef MH, Sherman G, Ward S, Fraser VJ (1999). Inadequate antimicrobial treatment of infections: a risk factor for hospital mortality among critically ill patients. Chest.

[11] Falagas ME, Barefoot L, Griffith J, Ruthazar R, Snydman DR (1996). Risk factors leading to clinical failure in the treatment of intra-abdominal or skin/soft tissue infections. Eur J Clin Microbiol Infect Dis.

[12] WHO: Antimicrobial resistance: Global report on surveillance. In 2014. >http://www.who.int/drugresistance/documents/surveillancereport/en/. Accessed 21 Jan 2016.

[13] Edelsberg J, Berger A, Schell S, Mallick R, Kuznik A, Oster G (2008). Economic consequences of failure of initial antibiotic therapy in hospitalized adults with complicated intra-abdominal infections. Surg Infect.

[14] Leekha S, Terrell CL, Edson RS (2011). General principles of antimicrobial therapy. Mayo Clin Proc.

[15] Koningstein M, van der Bij AK, de Kraker ME, Monen JC, Muilwijk J, de Greeff SC, Geerlings SE, van Hall MA, Group I-AS (2014). Recommendations for the empirical treatment of complicated urinary tract infections using surveillance data on antimicrobial resistance in the Netherlands. PLoS One.

[16] Grabe M, Bjerklund-Johansen TE, Botto H, Cek M, Naber KG, Tenke P, Wagenlehner F. Guidelines on urological infections. In: European Association of Urology. 2010;

[17] Hsueh PR, Hoban DJ, Carmeli Y, Chen SY, Desikan S, Alejandria M, Ko WC, Binh TQ (2011). Consensus review of the epidemiology and appropriate antimicrobial therapy of complicated urinary tract infections in Asia-Pacific region. J Inf Secur.

[18] Zhanel GG, Chung P, Adam H, Zelenitsky S, Denisuik A, Schweizer F, Lagace-Wiens PR, Rubinstein E, Gin AS, Walkty A (2014). Ceftolozane/tazobactam: a novel cephalosporin/beta-lactamase inhibitor combination with activity against multidrug-resistant gram-negative bacilli. Drugs.

[19] Sader HS, Farrell DJ, Flamm RK, Jones RN. Ceftolozane/tazobactam activity tested against aerobic Gram-negative organisms isolated from intra-abdominal and urinary tract infections in European and United States hospitals (2012). J Infect. 2014;69(3):266-77. 10.1016/j.jinf.2014.04.004 24780763

[20] Basu A (2016). Estimating costs and valuations of non-health benefits in cost-effectiveness analysis, in chapter 8, second panel on cost-effectiveness analysis in health and medicine: Oxford university press; forthcoming.

[21] AMCP. The AMCP Format For Formulary Submissions Version 3.1. In; 2012. http://www.amcp.org/practice-resources/amcp-format-formulary-submisions.pdf. Accessed 21 Jan 2016.

[22] Wagenlehner FM, Umeh O, Steenbergen J, Yuan G, Darouiche RO (2015). Ceftolozane-tazobactam compared with levofloxacin in the treatment of complicated urinary-tract infections, including pyelonephritis: a randomised, double-blind, phase 3 trial (ASPECT-cUTI).

[23] MacVane SH, Tuttle LO, Nicolau DP (2014). Impact of extended-spectrum beta-lactamase-producing organisms on clinical and economic outcomes in patients with urinary tract infection. J Hosp Med.

[24] United States Life Tables http://www.cdc.gov/nchs/products/life_tables.htm. Accessed 21 Jan 2016.

[25] 2012 Healthcare Cost and Utilization Project (HCUP). https://hcupnet.ahrq.gov/. Accessed 10 Nov 2014.

[26] Analy$ource Suite of Drug Pricing Services https://www.analysource.com. Accessed 21 Jan 2016.

[27] Using appropriate price indices for analyses of health care expenditures or income across multiple years. http://meps.ahrq.gov/about_meps/Price_Index.shtml. Accessed 21 Jan 2016.

[28] Jansen JP, Kumar R, Carmeli Y (2009). Cost-effectiveness evaluation of ertapenem versus piperacillin/tazobactam in the treatment of complicated intraabdominal infections accounting for antibiotic resistance. Value in health: the journal of the International Society for Pharmacoeconomics and Outcomes Research.

[29] Zerbaxa U.S. Prescribing Information [http://www.merck.com/product/usa/pi_circulars/z/zerbaxa/zerbaxa_pi.pdf]. Accessed 21 Jan 2016.

[30] Framework Summary [http://icer-review.org/wp-content/uploads/2016/02/Value-Assessment-Framework-One-Pager.pdf].

[31] Marchaim D, Gottesman T, Schwartz O, Korem M, Maor Y, Rahav G, Karplus R, Lazarovitch T, Braun E, Sprecher H (2010). National multicenter study of predictors and outcomes of bacteremia upon hospital admission caused by Enterobacteriaceae producing extended-spectrum beta-lactamases. Antimicrob Agents Chemother.

[32] Aloush V, Navon-Venezia S, Seigman-Igra Y, Cabili S, Carmeli Y (2006). Multidrug-resistant Pseudomonas Aeruginosa: risk factors and clinical impact. Antimicrob Agents Chemother.

[33] Sader HS, Mallick R, Kuznik A, Fritsche TR, Jones RN (2007). Use of in vitro susceptibility and pathogen prevalence data to model the expected clinical success rates of tigecycline and other commonly used antimicrobials for empirical treatment of complicated skin and skin-structure infections. Int J Antimicrob Agents.

[34] Lin HA, Yang YS, Wang JX, Lin HC, Lin Y, Chiu CH, Yeh KM, Lin JC, Chang FY. Comparison of the effectiveness and antibiotic cost among ceftriaxone, ertapenem, and levofloxacin in treatment of community-acquired complicated urinary tract infections. J Microbiol Immunol Infect. 2015; 10.1016/j.jmii.2014.12.010 25661278

[35] 2013 Healthcare Cost and Utilization Project (HCUP). [http://hcupnet.ahrq.gov/].

