# Supplementary material for: Cost-effectiveness of ceftolozane/tazobactam compared with piperacillin/tazobactam as empiric therapy based on the in-vitro surveillance of bacterial isolates in the United States for the treatment of complicated urinary tract infections
Source: BMC Infect Dis. 2017 Apr 28;17:314. doi: 10.1186/s12879-017-2408-7 (PMC5410095; doi:10.1186/s12879-017-2408-7)
Supplement: Additional file 1: — Details regarding PACTS and Premier data. This additional file provides readers with additional details regarding the Program to Assess Ceftolozane/Tazobactam Susceptibility (PACTS) dataset which was used to provide susceptibility inputs for the model and also regarding the Premier research database which was used to help define the pathogen distribution in cUTI. (DOCX 29 kb) [file 12879_2017_2408_MOESM1_ESM.docx]

**Additional file 1**

Susceptibility inputs - Program to Assess Ceftolozane/Tazobactam Susceptibility (PACTS) dataset

The isolates used for the patient-level simulation were sampled from the PACTS surveillance data. PACTS is a surveillance study evaluating the activity of ceftolozane/tazobactam and numerous anti-gram-negative agents against contemporary clinical isolates obtained from hospitalized patients with serious infections including bloodstream infections, skin and skin structure infections, urinary tract infections, intra-abdominal infections, and respiratory tract infections.^1^ The isolates were consecutively collected from 28 medical centers located in the US and 31 medical centers in 15 European countries from January 2011 to December 2013.^2^ One isolate per patient infection was included in the surveillance. Only isolates obtained from US sites were included in the analysis.

To assess the antimicrobial activity of ceftolozane/tazobactam and other (comparator) agents, the isolates were tested for susceptibility to these agents. Minimum inhibitory concentration (MIC) values of the agents were determined using standard broth micro-dilution methods as described by the Clinical and Laboratory Standards Institute (CLSI).^3^ In the model, susceptibility to empiric therapy is based on the measured susceptibilities reported in the PACTS dataset.

The *in vitro* surveillance data from PACTS represents the best available, patient level, real-world data reflecting cUTI patients at risk of resistant infection in the US. However, it is not possible to exclude non-complicated UTI infections, with the exception of isolates specifically noted to be catheter-related, within PACTS. Therefore, the PACTS dataset may not reflect the distribution of pathogens most likely to cause cUTI. For this reason, the Premier study was used to estimate a pathogen distribution specific to cUTI.

cUTI pathogen distribution – Premier database

Premier is an alliance of community-based hospitals with over 2,800 hospital members.^4^ The database is a complete census of inpatients and hospital-based outpatients from geographically diverse hospitals in the US. The database currently contains data from more than 480 million patient encounters, or one in every five discharges in the US. Laboratory results are available from a subset of facilities in the Premier research database.

An algorithm based on a set of ICD-9 diagnosis codes and current procedural terminology (CPT) procedure codes was used to identify cUTI patients from the Premier database between January 1, 2009 and March 31, 2013. The cUTI cohort consisted of 251,707 urinary isolates, with the pathogen distribution described as: 60.4% *E. coli*, 16.4% *K. pneumoniae*, 9.1% *P. aeruginosa*, and 0.8% *Acinetobacter spp*. Most patients with positive cultures were elderly, the mean age was 59±19 years (median age: 75 years). The sum of the pathogens exceeds 100% because patients could have polymicrobial infection. Because the model only considers monomicrobial infection, the Premier distributions were normalized to 100%.

Although the Premier database represents a large isolate source, it was not possible to conduct the entire analysis using the Premier database because the isolates were not tested for susceptibility to ceftolozane/tazobactam. Therefore, data from the Premier database was used in conjunction with the PACTS dataset in the model.

**References**

1. Farrell DJ. *Surveillance of ceftolozane/tazobactam antimicrobial activity tested against gram-negative organisms and streptococci (selected) isolated in the United States (13-CUB-02-USA/CXA.087.MC).* Cubist Pharmaceuticals;2013.

2. Sader HS, Farrell DJ, Flamm RK, Jones RN. Ceftolozane/tazobactam activity tested against aerobic Gram-negative organisms isolated from intra-abdominal and urinary tract infections in European and United States hospitals (2012). *The Journal of infection.* 2014;69(3):266-277.

3. Clinical and Laboratory Standards Institute M07-A9. Methods for dilution antimicrobial susceptibility tests for bacteria that grow aerobically; approved standard. *CLSI.* 2012;9th edition Wayne.

4. Premier database. <https://www.premierinc.com/>. Accessed December 8, 2015.
